# Pulmonary Embolism and Severe Asthma: Case Report and Literature Review

**DOI:** 10.3390/medicina55100647

**Published:** 2019-09-26

**Authors:** Po-Hsin Lee, Pin-Kuei Fu

**Affiliations:** 1 Department of Internal Medicine, Taichung Veterans General Hospital, Taichung 40705, Taiwan; berry7bo@gmail.com; 2 Department of Critical Care Medicine, Taichung Veterans General Hospital, Taichung 40705, Taiwan; 3 College of Human Science and Social Innovation, Hungkuang University, Taichung 43302, Taiwan; 4 Science College, Tunghai University, Taichung 40704, Taiwan

**Keywords:** severe asthma, coagulopathy, massive pulmonary embolism, plasminogen activator inhibitor type I (PAI-1), tissue factor (TF)

## Abstract

Pulmonary embolism is a life-threatening disease. Its development is generally thought to be due to causes collectively known as the Virchow’s triad. Chronic inflammations are associated with the activation of coagulation and increased risks of venous thromboembolic events. Asthma is one of the chronic inflammatory diseases associated with procoagulants and antifibrinolytic activities in the airways. Coagulation is activated in patients with asthma with the following steps of pathophysiology: Increased tissue factor expression in various cell types, decreased activity of the anticoagulant protein C system and inhibition of fibrinolysis through over-production of plasminogen activator inhibitor type 1 (PAI-1). Asthma is therefore likely a risk factor for pulmonary embolism, especially in those patients with severe disease conditions together with frequent exacerbation. Here we present a case of severe asthma associated with coagulopathy and complicated by massive pulmonary embolism, presented with typical S1Q3T3 on electrocardiography (ECG) and massive thrombosis on computed tomography angiography, successfully treated with directed catheter thrombolytic therapy.

## 1. Introduction

Pulmonary embolism, a blockage of arteries in the lung, is a life-threatening disease. The development of pulmonary embolism is classically considered due to risk factors named Virchow’s triad (alterations in blood flow; factors associated with endothelium damage in the vessel wall; and factors affecting blood properties). Increasing evidence suggests that asthma is associated with greater risks of pulmonary embolism [1,2,3,4,5]. Here, we report a case of a 62-year-old female who, having been diagnosed with severe asthma, had developed recurrent pulmonary embolism. We also reviewed the related studies in the literature and proposed mechanisms on how asthma had activated coagulation.

## 2. Case Presentation

**Figure 1:**
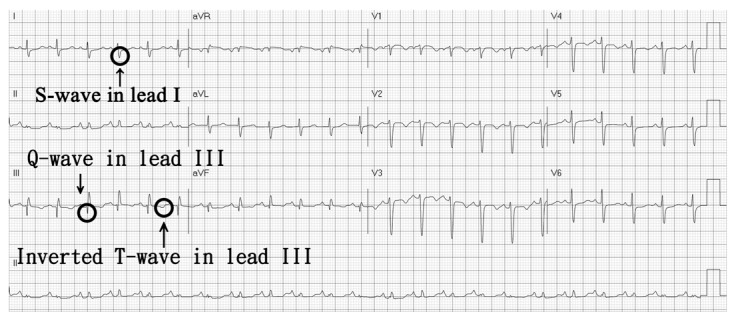
Electrocardiography-revealed S1Q3T3 pattern.

**Figure 2:**
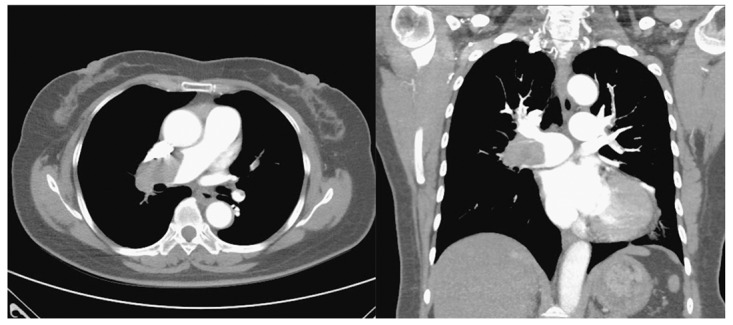
Computed tomography angiography demonstrates massive thrombosis in bilateral pulmonary arteries, predominantly right-sided.

A 62-year-old female with a medical history of asthma was brought to the emergency department for a week-long symptom of shortness of breath. According to her medical records, she had been diagnosed with pulmonary embolism and deep vein thrombosis 13 years ago. She was then treated with enoxaparin and warfarin and was later discharged with regular followed up in our cardiovascular outpatient clinic. Asthma was diagnosed based on an obstructive airflow limitation accompanied by positive bronchodilator test and symptoms. Her asthma was controlled by long-term medications of Symbicort Turbuhaler, two puffs twice per day, and Spiriva Respimat, two puffs per day according to the Global Initiative for Asthma (GINA) guideline. This time, she suffered from shortness of breath for a week and returned to our emergency department. Physical examinations showed tachycardia (101/min) and hypertension (173/104), and her body temperature was 37.4 °C. Her D-dimer level was 2.64 mg/L and troponin level was within the normal range. Electrocardiography revealed sinus tachycardia, and S1Q3T3 pattern was recorded (Figure 1). No abnormal findings were obtained in her chest roentgenography. Computed tomography angiography revealed thrombosis in bilateral pulmonary arteries, corresponding to massive pulmonary embolism (Figure 2). Rivaroxaban was administered but her symptoms did not improve. As a result, she had received catheter-directed thrombolytic therapy by Ekosonic Endovascular System (EKOS, BTG International Ltd., London, United Kingdom), together with recombinant tissue plasminogen activator infusion. Symptoms thereafter were relieved and followed-up pulmonary artery angiography showed decreased thrombus burden and a drop in the pulmonary arterial pressure from 68 to 40 mmHg. We surveyed the etiology of recurrent pulmonary embolism including the following: Tumor markers, antinuclear antibodies, anticardiolipin antibodies, anti-beta 2 glycoproteins, lupus anticoagulant, protein C, protein S, antithrombin III, and the results were all were within normal ranges. Abdominal ultrasound also revealed no abnormality. The patient was then discharged under long-term medication of rivaroxaban. Written informed consent was obtained from the patient for this study.

## 3. Discussion

Pulmonary embolus (PE) refers to the obstruction of a pulmonary artery or its branches by some material (e.g., thrombus, tumor, air, or fat). Inherited and acquired risk factors exist that cause the development of venous thrombosis. Common causes of an inherited hypercoagulable state include the following: Factor V Leiden; prothrombin gene mutations; and defects in protein S, protein C, or antithrombin [6,7]. Acquired risk factors include prior thrombotic event, recent major surgery, central venous catheter indwelling, trauma, immobilization, malignancy, pregnancy, medications (e.g., oral contraceptives), antiphospholipid syndrome, and chronic inflammation [3,4,6,8].

**Figure 3:**
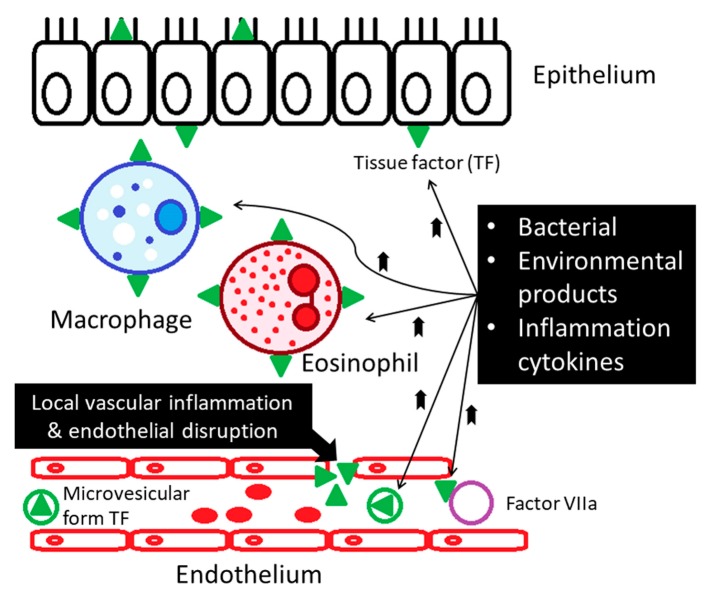
Increased levels of tissue factor are found in patients with asthma. Local vascular inflammation and endothelial disruption contribute to procoagulant state in the asthmatics.

Asthma is one of the chronic inflammatory diseases associated with procoagulants and antifibrinolytic activities in the airways. In patients with asthma, hyper-coagulation is activated through mechanisms of (1) increased expression of the tissue factor (TF), (2) decreased activity of anticoagulant protein C (PC) system, and (3) inhibition of fibrinolysis through over-production of plasminogen activator inhibitor type 1 (PAI-1) [4,9,10,11]. Tissue factor (TF) is considered the main initiator of coagulation, which binds and activates clotting factor VII (FVII), subsequently generating factor FVIIa. In chronic asthmatic patients, increased levels of tissue factor (TF) are found due to local vascular inflammation, and endothelial disruption contributes to procoagulant state [2,9,12]. By the stimulation of proinflammatory cytokines (such as IL-6 and TNF-α), TF becomes exposed on the surface of macrophages, eosinophils, epithelial, and endothelial cells [2,9,12] (Figure 3). The airway of asthmatic patients is also characterized by microvascular hyperpermeability, which could contribute to localized procoagulant state and the subsequent thrombus formation [11] (Figure 3).

**Figure 4:**
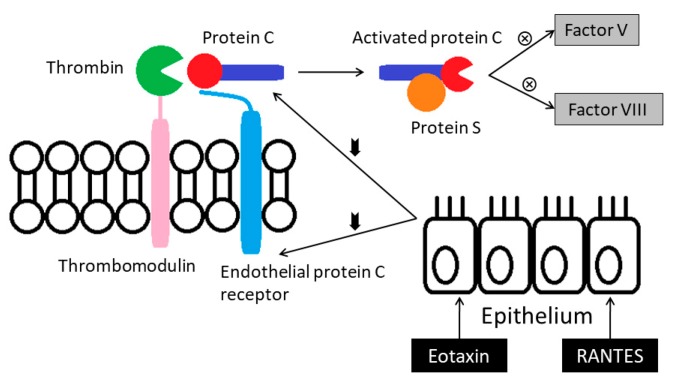
Activities of the protein C anticoagulant system appear lower in the asthmatics. RANTES: Regulated on activation, normal t-cell expressed, and secreted.

Activities of the protein C anticoagulant system appear lower in the asthmatics. The system is activated when thrombin binds with thrombomodulin that converts protein C (PC) to the activated form. This activation is augmented by the endothelial protein C receptor (EPCR). The suppressed protein C pathway in the asthmatics could be the result of down-regulation of PC and EPCR. Bronchial epithelial cells exposed to eotaxin or RANTES (regulated on activation, normal t-cell expressed, and secreted) in vitro reduce their expressions of PC and EPCR mRNA [13] (Figure 4).

**Figure 5:**
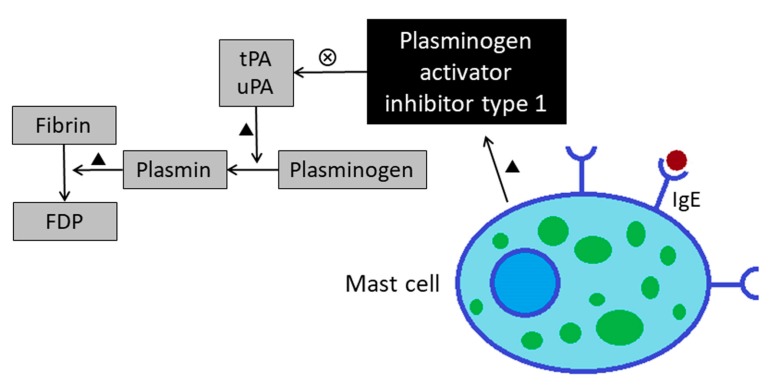
Increased levels of plasminogen activator inhibitor type 1 leads to impaired fibrinolysis function in asthmatics. FDP: Fibrinogen degradation product; tPA: Tissue plasminogen activator; uPA: Urokinase-type plasminogen activator.

Fibrinolysis is the process of breaking down the fibrin clot, and this process is conducted by plasmin. Tissue-type plasminogen activator (tPA) and urokinase-type plasminogen activator (uPA) are the agents that convert plasminogen to the active plasmin. Plasminogen activator inhibitor type I (PAI-1) is the major inhibitor of both tPA and uPA. Mast cells are probably an important source for PAI-1 in the asthmatic lung. Lung tissues of patients with asthma and rats challenged with ovalbumin displayed more PAI-1–positive mast cells [14]. In addition, transcription of PAI-1 is markedly up-regulated in human mast cells in vitro when stimulated with IgE [15] (Figure 5).

We summarize the researches of investigating risk of pulmonary embolism in asthmatic patients in Table 1 [1,4,6,7,8,10]. An early cohort study analyzed risk factors for venous thromboembolism; however, no association between asthma and either deep vein thrombosis (DVT) or PE was found in this cohort [7]. Majoor et al. reported that severe asthma is an independent risk factor for PE, but not for DVT [6]. Two nationwide cohort studies conducted by Chung et al. [8] and Yeh et al. [10] showed that asthmatic patients or those with ACOS (asthma-chronic obstructive pulmonary disease overlap syndrome) would have higher risks of pulmonary embolism [8,10]. In contrast to the findings of Majoor et al., Zöller et al. found an increased risk in both PE and DVT in asthma patients, a discrepancy in results likely related to their larger sample size [1]. In summary, the incidence rate of pulmonary embolism in asthma patients may increase with higher disease severity, increased frequencies of asthma exacerbation and hospitalization and the uses of inhaled or systemic steroids, as in previous researches.

For the diagnosis of pulmonary embolism, electrocardiography (ECG) is also a valuable tool in prognostication of acute PE. In a systematic review and meta-analysis, specific features in the ECG are predictive of in-hospital deaths. Such features are S1Q3T3, complete RBBB, TWI, ST-segment depression in V4 through V6, STE-V1, STE-III, Qr-V1, RAD, AF, and RV transmural ischemic pattern [16]. 

Our patient required high doses of inhaled corticosteroids/long-acting beta-agonist plus long-acting muscarinic antagonist for asthma control, which was classified as severe asthma according to GINA guideline. Therefore, severe asthma needing inhaled corticosteroids (ICS) or oral corticosteroids (OCS) might be a risk factor for her pulmonary embolism. The massive pulmonary embolism was diagnosed by the typical S1Q3T3 pattern in the ECG and, computed tomography angiography revealed thrombosis in bilateral pulmonary arteries. As the S1Q3T3 pattern might indicate a poor prognosis, she received catheter-directed thrombolytic therapy, which was reasonable in such condition, and the outcome was favorable.

## 4. Conclusions

Pulmonary embolism is a life-threatening disease. Asthma is one of the chronic inflammatory diseases associated with procoagulants and antifibrinolytic activities in the airways, which increases risks of venous thromboembolic events. Our case provides important information for clinicians that asthma is a risk factor for pulmonary embolism, especially in those patients with severe disease conditions together with frequent exacerbation. Hemodynamic patterns, right ventricular functional status, and specific features on ECG (e.g., S1Q3T3) might indicate a poor prognosis, and more invasive procedures should be considered in managing such patients. Clinicians should pay attention to evaluate the possibility of pulmonary embolism when dealing with chronic asthmatic patients developing an acute onset of dyspnea.

## Figures and Tables

**Table 1:** Previous studies reported increased risks of pulmonary embolism in patients with asthma.

Study Group	Methodology	Case Number	Result
Huerta et al. [7]	Cohort study	3544 DVT; 3066 PE; 10000 Control	DVT (OR 1.15, 1.00–1.31); PE (OR 1.32, 1.15–1.53)
Majoor et al. [6]	Retrospective	648 Asthma (severe: 283, mild to moderate: 365)	Severe asthma: HR = 3.33 (1.16–9.93); Corticosteroid use: HR = 2.82 (1.09–7.30)
Chung et al. [8]	Cohort study	31356 Asthma; 125157 Control	PE: HR = 3.24 (1.74–6.01); Increased risk with frequent asthma exacerbation and hospitalization.
Barreto et al. [5]	Retrospective	133 acute PE; (12% Asthma)	PE (OR 2.16, 1.26–3.71)
Yeh et al. [10]	Cohort study	ACOS: 14150; non-ACOS: 55876	ACOS for PE: HR = 2.08 (1.56–2.76) Using ICS: HR =1.97 (1.29–3.01); Using OCS: HR = 1.97 (1.46–2.65)
Zöller et al. [1]	Cohort study	PE: 114366; DVT: 76494; Both PE and DVT: 6854	PE (OR 1.43, 1.37–1.50); DVT (OR 1.56, 1.47–1.65); either PE or DVT (OR 1.60, 1.32–1.93)

PE: Pulmonary embolism; DVT: Deep vein thrombosis; ACOS: Asthma-COPD overlap syndrome; HR: Hazard ratio; ICS: Inhaled corticosteroids; OCS: Oral corticosteroids; OR: Odds ratio; OPD: Outpatient department.
